# From distance to embodiment—objectivity and empathy in Swedish rape trials

**DOI:** 10.3389/fsoc.2025.1461018

**Published:** 2025-05-13

**Authors:** Moa Bladini

**Affiliations:** Department of Law, School of Business, Economics and Law, University of Gothenburg, Gothenburg, Sweden

**Keywords:** law and emotions, criminal law, empathy, objectivity, rape trials, standpoint epistemologies, himpathy and herasure, male and female fear

## Abstract

This article investigates how objectivity is performed and embodied in Swedish rape trials, where legal decisions often hinge on oral testimonies rather than technical evidence. Drawing on the sociology of emotions and feminist legal theory, the article challenges the positivist notion of objectivity as dispassionate detachment. Instead, it conceptualizes objectivity as a situated and emotionally regulated practice, co-produced through empathic translation and imagination. Based on ethnographic fieldwork—including observations, interviews with legal professionals, and analysis of 18 rape cases—the study shows how empathy serves as a critical epistemic tool in the courtroom. Judges and other legal actors must translate everyday experiences into legal logics while maintaining impartiality. Concepts such as himpathy and herasure (Manne), female fear, and male fear are used to explore how gendered norms shape credibility assessments and emotional orientations in rape trials. The article argues that empathy does not undermine objectivity, but rather constitutes its condition in cases where normative assumptions and lived experiences diverge. Harding's standpoint epistemology and concept of strong objectivity inform a model of legal reasoning that is reflexive, perspectival, and emotionally attuned. The study identifies how empathic trials—where legal actors actively engage with gendered perspectives—can counteract testimonial and hermeneutical injustice, thus fostering more equitable adjudication. Ultimately, the article advocates for a reconceptualisation of objectivity as embodied and relational, particularly crucial in the legal treatment of sexual violence.

## Introduction

In the framework of liberal democracies, the judicial system stands as a pillar of governance, securing its public credibility through its commitment to impartial and transparent administration of justice. The principle of the rule of law ensures that all individuals are treated equally before the law^1^.

Criminal law represents one of the most intrusive forms of state authority; thus, it is essential that criminal trials are both conducted fairly and seen to be fair, with judges maintaining impartiality to ensure justice for everyone. The question of objectivity has traditionally been dealt with as an ideal, embedded in the modern positivist ideal, particularly so in a legal scholarly debate (e.g., Rawls, 1999; Weber, 1998; Maroney, 2011; Nedelsky, 2011; Bladini, 2013). This ideal may be illustrated by Rawls (1999)' ideal judge placed under the “veil of ignorance” to become detached from context and relationships. The objectivity ideal is critical for securing societal trust and upholding the legitimacy of the judiciary. Objectivity has, in line with a positivist tradition, been understood as factuality, impartiality, and being achieved solely through reason, meaning being dispassionate and without engagement with emotions (Maroney, 2011; Bladini, 2013).

However, objectivity has, by scholars from various scientific fields, been showed to be far from unemotional and disembodied (Bladini, 2013; Bergman Blix and Wettergren, 2018; Lynch et al., 1995; Latour, 2002; Scheffer, 2010; van Oorschot, 2021). Bergman Blix and Wettergren (2018) highlight how judges often display objectivity as a non-emotional state, yet this display is the product of a sophisticated process of managing both their own and others' emotions. Lynch's research has demonstrated how scientific facts, truths, and objectivity are not merely discovered, but are actively constructed and *performed* through embodied practices in legal contexts (Lynch et al., 1995). This resonates with Latour's *actor-network theory* (ANT), which similarly challenges the notion of truth and objectivity as static or absolute, instead positioning them as the products of complex networks of human and non-human actors, material processes, and social practices (Latour, 2002).

Cases involving sexual violence in general, and rape in particular, challenge the traditional ideal of objectivity to its core. In these cases, lived experiences of victims and defendants, as well as embodied experiences, emotions and shared memories of legal actors are at play. Trials in these cases often focus on oral evidence, making the courtroom a site where empathy and objectivity intersect (Bladini et al., 2023; Wettergren et al., 2025).

Sweden, a hybrid legal system, as part of the Scandinavian legal culture, is internationally highly ranked in gender equality^2^, and has a relatively newly implemented consent-based rape law (in 2018). Yet, there are challenges, and the legislation on sexual violence is the part of Swedish criminal law that has undergone the most reforms (Träskman and Wennberg, 2019) and consistently provokes criticism—both from those advocating for harsher penalties or broader legal provisions and from those who argue that its complexity undermines legal certainty (Leijonhufvud, 2015; Proposition, 2017/18:177). Despite extensive reforms, significant challenges remain, particularly when it comes to its application. The number of solved cases and convictions has historically been and still remain relatively low, compared to many other types of crime (Brå, 2025: p. 3). Moreover, the application of the law has frequently been criticized for being distressing or degrading for victims (Leijonhufvud, 2015; Proposition, 2017/18:177). Therefore, Swedish rape trials are of particular interest when scrutinizing the objectivity ideal and practice in criminal legal procedure.

This article examines how objectivity is performed in Swedish rape trials, with particular attention to the intersection of empathy and objectivity in judicial decision-making. The analysis draws on a combination of the sociology of emotions—focusing on concepts such as empathy, empathic translation and empathic imagination—and feminist theory, including notions such as himpathy, herasure, and male and female fear.

The aim is 2-fold: 1) to demonstrate how empathy operates as a tool for judges and other legal actors to translate or imaginatively relate everyday life experiences to legal logics in the performance of objectivity, and 2) eluminate how standpoint perspectives—especially feminist accounts such as the concept or female fear can serve as critical resource for empathic translation and imagination, thereby countering the effects of himpathy and herasure in legal contexts.

The article starts with a brief introduction to the Swedish legal context, followed by a short description of the theoretical and methodological framework including the material, then the analysis is presented in the section *Objectivity in Practice—Empathic translations and dispassionate encoding* and the article ends with concluding remarks.

## The Swedish legal context

The legal systems of Scandinavia are frequently described as hybrid systems, founded primarily on codified law, yet incorporating aspects of prior case law. Situated between the civil law tradition of continental Europe and the common law system, these legal systems have historically, and in substance, demonstrated a closer alignment with the continental legal tradition than with common law (Bogdan and Wong, 2022: p. 10).

A key foundational principle within Swedish courts and government institutions is the principle of transparency and public access, which ensures that the public can access official records and, for instance, attend criminal trials (except when closed sessions are necessary to protect conflicting interests, which is regularly the situation in rape cases; Bogdan and Wong, 2022: p. 14).

### Objectivity regulated

Objectivity in Swedish courts is regulated by sets of rules with different functions: establishing rules and granting rules. The first set of constituent rules establish a requirement that criminal proceedings be conducted objectively. The requirement of objectivity as expressed in the Constitution (RF 1:9; 2:11)^3^, and in the European Convention of Human Rights (ECHR) and the Treaty of Lisbon's reference to it, which deals with the right to a fair trial.^4^ The judicial oath can be understood as a mechanism that instills the requirement of objectivity not merely as an abstract principle, but as an embodied commitment in the professional conduct of every incoming judge (RB 4:11).^5^

In jurisdictions governed by the rule of law, merely establishing a requirement for objectivity in judicial activities is insufficient. The rules must also perform an executive function. The mechanisms designed to enforce judicial objectivity in Sweden can be categorized into two distinct groups: rules of competence and procedural rules. The former includes regulations concerning the independence of judges, their qualifications, and their ability to adjudicate. This category also covers provisions for the removal of judges from office, and oversight of their activities, including criteria for disqualification in specific cases. The latter group, procedural rules, addresses the conduct of criminal proceedings, the assessment of evidence, and accountability measures. Together, these rules form a comprehensive framework intended to uphold the integrity and impartiality of the judiciary (Bladini, 2013).

Striking for these both sets of rules are that they focus on the distance between the judge and the parties, and that the judiciary must be *seemingly* objective, but they lack discussion on *how* the requirement of objectivity shall be fulfilled.

### The criminal procedure and rape legislation

The Swedish criminal procedure is based on adversarial and negotiation principles, structured around two opposing parties, and predominantly accusatorial in nature. However, the judge may assume an active role to ensure the thorough investigation of the case (Ekelöf and Edelstam, 2002). The only procedural element that is legally binding for the court is the description of the criminal act as stated in the charge, which also sets the parameters for the trial (RB 30:3).^6^ The process is governed by the foundational principles of free admissibility and free evaluation of evidence (Ekelöf et al., 2009; Fitger, 2014; Holmgård, 2019).^7^ The burden of proof rests with the prosecutor, and the standard of proof is set to a high threshold, i.e., the guilt must be proven beyond reasonable doubt (Ekelöf and Edelstam, 2002). Several key principles underpin Swedish criminal procedure: the principle of orality (cf. Bylander, 2006), the principle of immediacy (which requires that only evidence presented during the trial may be considered), and the principle of concentration, which stipulates that the trial should be conducted within a concentrated time frame (Wong, 2012; Ekelöf et al., 2009). Finally, as mentioned above, a fundamental principle worth mentioning is the principle of transparency and public access to judicial proceedings, which means that courtrooms are open to the public, including during criminal trials. However, due to the high sensitivity of rape trials—for instance, the classified nature of the proceedings and the confidentiality measures taken to protect the victim—such trials are usually held behind closed doors.

The Swedish rape legislation has gone through many reforms, the latest one in 2018 when the explicit requirement of non-voluntariness was introduced, together with the new crime, negligent rape. This is one of many consent-based models introduced in Europe and elsewhere, and the Swedish model falls under the affirmative consent model (Uhnoo et al., 2024a; Wegerstad, 2021). The legal change has been met with both hope and concern, and research indicates that while some of the intended effects of the reform have been achieved, the shift in norms is slow, and several challenges remain (Wettergren et al., 2025; Brå, 2025).

### The legal professionals and parties in criminal cases

In Swedish trials the court consists of one legal judge and three lay judges in District Court, and three legal judges and two lay judges in Court of Appeal. All judges, legal and lay, have equal votes.^8^ In the event of a tie in votes, the decision that results in the most lenient outcome for the defendant prevails.

Apart from the judges, three other legal professionals participate in the trials: the prosecutor, the defense lawyer, and the victim's counsel.

The prosecutor is bound by the principle of objectivity, not only during the pre-investigation, but also during the trial (RB 23:4 & 45:3 a).^9^ A keyway to understand how prosecutors interpret and perform their role in court is hence through the influence of the objectivity principle. Prosecutors also appear to assume that facts speak for themselves. In our previous studies, many of the prosecutors seemed to rely on the assumption that the court, particularly the legally trained judges, can independently assess and interpret the presented facts without the need for extensive framing or contextualization. This reflects an underlying belief that judges, as legal experts, are capable of drawing conclusions from the evidence without significant narrative guidance from the prosecution (Bladini et al., 2023).

However, this approach may be less effective in rape cases, where the primary evidence typically consists of oral testimonies. In such cases, the nature of the evidence may demand a more nuanced presentation, compared to cases involving complex technical data, where prosecutors are generally more diligent in framing and explaining the facts. Nonetheless, there are some prosecutors who challenge this traditional objectivity, and perform a more active role in shaping the narrative, in line with a collectively embodied objectivity.

The defense lawyer is not bound by any ideal of objectivity, but rather represents a client and may therefore adopt a fully partial stance. Defense lawyers are often skilled in the rhetorical framing of evidence and are acutely aware that their efforts may influence the outcome of the case. In particular, they engage in strategic rhetorical and emotional framing (Wettergren et al., 2025), an aspect further explored below in the theoretical point of departure.

The victim's counsel is appointed by the court to support victims of serious crimes, such as sexual and violent offenses, by ensuring that their rights and interests are represented throughout the judicial process. This role involves providing essential legal advice and emotional support, thereby assisting complainants in navigating the complexities of the criminal justice system (Proposition, 1987/88:107; NJA II, 1988). Responsibilities typically include advising on legal matters, supporting the preparation and presentation of evidence, and advocating for the victim's rights, particularly in relation to compensation (Tham et al., 2011).^10^ However, victim's counsel are often relatively junior lawyers, and since the prosecutor holds the primary responsibility for the criminal case, the remit of the victim's counsel can become indistinct—particularly concerning her capacity to mandate to engage with issues of guilt. At times this position has been characterized as operating in the shadow of the prosecution, relying heavily on the prosecutor's lead rather than pursuing an independent legal strategy.

The complainant is in general present during District Court (DC) proceedings but not during Appellate Court (AC) hearings. In the latter, video-recorded testimonies from the DC trial are used, eliminating the need for new examinations. The AC reviews these recordings instead of conducting new interrogations, which is considerate of the complainant's wellbeing, reducing additional psychological distress. This procedural distinction highlights the Swedish judicial system's compassionate approach, prioritizing the mental wellbeing and comfort of the complainant while maintaining the integrity of the legal process through recorded testimony. However, following the introduction of Sweden's consent-based rape legislation, the victim's counsel's right to be present in court during trial has been curtailed, now requiring special reasons for participation (Proposition, 2017/18:86). This shift introduces a potential imbalance in how the parties appear before the court.

While the defendant and defense lawyer are present, the complainant and her counsel is not.

## Theoretical point of departure and methodological framework

The article builds on two strands of research: sociology of emotions and feminist theory. A fundamental understanding in this article is that emotions and cognitive reason are intertwined in legal decision making (de Sousa, 1987; Etzioni, 1988; Damasio, 1994; Barbalet, 1998). This is relevant for the understanding of objectivity, which is here understood, not as a state but a process, i.e., a doing of objectivity in practice. In this sense, the field of sociology of emotions is crucial. The embodiment of objectivity, inspired by Lynchs' work on embodied legal practice, will be scrutinized by combining Hardings standpoint epistemologies, and the emotive sociological concept empathy. One crucial point of departure is that objectivity is a constantly ongoing process where judges perform and do objectivity through an advanced work with emotion management, their own and others' as well as in cooperation with other legal actors in the court room, building on the work by Bergman Blix and Wettergren (2018).

This understanding will be combined with another objectivity ideal, that stems from feminist research, i.e., Hardings *strong objectivity* building on standpoint epistemologies. By combining empathy, a tool to understand someone else's perspective in the sociology of emotions theory, with two specific standpoint epistemologies, i.e., a female and male perspective of rape respectively, I will explore the challenges specific for rape cases mentioned above. The following concepts from feminist theory will also be used: *himpathy* and *herasure* as developed by Manne (2018), alongside the feminist concept of *female fear* which will be used together with the corresponding concept of *male fear*.

### Sociology of emotions and empathy

Emotions are traditionally viewed as incongruous with such judicial processes. Nonetheless, it is widely acknowledged that a criminal courtroom is inherently laden with emotions: a nervous witness, a frustrated suspect, and an anxious victim, with testimonies that often move listeners to tears. Despite this, the judge is expected to maintain an impassive demeanor and remain neutral (Bergman Blix and Wettergren, 2018). The prevailing notion is that emotions are irrelevant to the role of a judge and can therefore be set aside. This belief is attached to the positivist ideal of objectivity, which posits that emotions and reason are antithetical. Consequently, emotions are perceived as intrusions that disrupt the rational processes of conducting trials, evaluating evidence, and engaging in deliberations. However, research in philosophy, neuroscience, social psychology, and sociology has demonstrated that emotions and reason are intertwined and collaboratively facilitate rational decision-making (e.g., Nussbaum, 1996; Damasio, 1994; Barbalet, 1998; Ask and Granhag, 2007; de Sousa, 2008).^11^

The expectations for how different actors express and manage their emotions can vary within the same context. Despite the deeply tragic testimonies that may affect everyone in the courtroom, judges have developed strategies to manage their emotions, ensuring these do not compromise their objectivity. Background emotions play a vital role in the knowledge-seeking process and are crucial for legal decision-making. Such emotions, integral to the pursuit of knowledge, are known as epistemic emotions. Epistemic emotions are essential to cognitive processes, providing information and motivating mental action (Arango-Muñoz, 2014), they contribute to knowledge acquisition by guiding attention, motivating action, and supplementing reason (de Sousa, 2008; Barbalet, 1998; Nussbaum, 1996). Commonly recognized epistemic emotions include certainty, understanding, curiosity, epistemic anxiety, and uncertainty (de Sousa, 2008). The feeling of not knowing is particularly prevalent among judges, and other legal actors in rape cases. How this, and other emotions unfold in rape trials are further explored by Wettergren et al. (2025).

In a courtroom, legal actors follow an emotional regime that views emotions as disruptive to rationality and thus should be suppressed. However, in rape trails, it could be noted that male fear and himpathy are strongly embedded, intertwined with an anxiety of being biased with the complainant and to convict someone innocent (Uhnoo et al., 2024b; Wettergren et al., 2025).

Empathy is not an emotion like sympathy or compassion but a capacity to attune to others' emotions (Bandes, 2009; Basch, 1983). It involves imagining how others experience the world. Nussbaum suggests that judges should read a case as if reading a novel, employing empathetic identification alongside critical assessment (Nussbaum, 1996). Similarly, Del Mar advocates using imagination to understand different perspectives (Del Mar, 2017). While studies in the legal field have highlighted empathy as a potential source of bias (Fisher, 1987; Bandes, 2009), it remains a crucial tool for gathering information about a case. Judges need to use empathy, in terms of empathic imagination, to understand the actions and perspectives of parties and witnesses, and to evaluate credibility and trustworthiness. Additionally, other legal actors must be empathetic translators in court, converting the fuzzy everyday life narratives that needs to embody legal concepts into legal logic. Hence, they need to work with empathic translation. However, this process of encoding lay narratives into legal frameworks is tied to the requirement of dispassion (cf. Törnqvist, 2021; Bladini et al., 2023; Wettergren et al., 2025; Bergman Blix and Minissale, 2022).

### Feminist theory

*Harding critiques the positivist view* of objectivity, calling it “weak objectivity” because it overlooks the values and perspectives taken for granted within the scientific community (or among judges). These underlying assumptions are embedded in the structures, practices, and language of science, and thus become invisible under traditional positivist objectivity, appearing as natural and necessary. Modern science, Harding argues, is shaped by certain values and interests, such as those of western, bourgeois, and patriarchal societies (Harding, 1992). She suggests that scientists—and by extension, judges—should critically examine their own values by considering perspectives from marginalized groups. This process, involving engagement with different perspectives, aligns with standpoint epistemologies (Harding, 1992). In legal contexts, Harding's strong objectivity and standpoint epistemologies can be linked to the concept of empathy, a key tool in the sociology of emotions, which allows individuals to understand others' perspectives and emotions (Basch, 1983; Bandes, 2009; Bergman Blix and Wettergren, 2016). In legal decision-making, especially in the presentation and evaluation of oral evidence like testimonies, empathetic imagination is essential. Objectivity in the courtroom is a collaborative effort, where the judge uses empathy to grasp the actions and perspectives of those involved. Other legal actors, such as defense lawyers and prosecutors, must translate clients' and witnesses' narratives into both legal logic and empathetic terms, bridging everyday life stories with legal concepts. This process of encoding lay narratives into legal logic is intrinsically linked to the requirement of dispassion (cf. Bergman Blix and Minissale, 2022; Bladini, 2013). This dual translation is vital for the judge to fully understand the reasoning and actions of the parties.

Harding's standpoint epistemologies may be of relevance for judges in their professional everyday life, in particular when understood in combination with the concept of empathy. To understand motives and intent, or the action rationality of complainants in rape cases, the judge needs empathic skills. They (judges) need to understand the perspective of the person that testifies before the court, and in this process the judge needs rather the embodied than the gods eye perspective.

The process of doing objectivity, as a situated emotive-cognitive process that necessitates emotional reflexivity (Törnqvist and Wettergren, 2023; Bergman Blix and Wettergren, 2018) may be connected to the objectivity ideal and standpoint epistemologies advocated by Harding, that includes the embodiment and empathic imagination.^12^ The empathic translation and imagination that needs to be done in rape trials are and may be influenced by himpathy, herasure, male fear and female fear.

The concepts of *himpathy* and *herasure* are developed by philosopher Manne (2018). Manne has investigated the structural and cultural mechanisms underpinning misogyny. She argues that misogyny is not merely a matter of individual attitudes or actions but rather a systemic phenomenon that sustains patriarchal power structures. She introduces the concepts of himpathy and herasure, to offer an explanation for how gender-related injustices and acts of violence are addressed in society and the legal system (Manne, 2018). These concepts shed light on structural and cultural phenomena that can influence legal processes and outcomes.

Himpathy (sympathy with him) refers to the disproportionately high level of sympathy and understanding often extended to men, particularly those accused of sexual violence. Manne describes how this sympathy can lead to perpetrators being treated with greater leniency and having their actions excused or rationalized. It is important to note that this is especially true for privileged men or men who generally conform to the image of being respectable individuals. In a legal context, himpathy can manifest through lighter sentences, reluctance to prosecute, or a tendency to question the credibility of victims. This undermines the legal system's ability to function fairly and may contribute to continued leniency or impunity for perpetrators (Manne, 2018).

Uhnoo et al. (2024b) describe how sympathies for men accused of sexual violence are closely tied to what they term *male fear*—a shared fear among men of being accused of sexual violence or assault. This fear paves the way for discourses shaped by himpathy, where sympathy for men accused of rape dominates, reinforced by concerns about wrongful convictions, the stigma of being labeled a rapist, or the potential destruction of their future. Such discourses are evident not only among defense lawyers but also among judges and prosecutors.

Himpathy permeates discussions about the legal handling of rape cases, and male fear is often strategically utilized by defense lawyers, appearing to influence judicial outcomes. While it is crucial in any legal system to ensure that no innocent person is convicted, in rape cases, the fear of false accusations seems to play a particularly prominent role, contributing to a system where few cases reach court and conviction rates remain lower than for other crimes (Manne, 2018; Brå, 2019, 2025; Uhnoo et al., 2024b).

The counterpart to male fear is *female fear*, a concept used by feminist scholars to explain women's behaviors and rationalizations aimed at avoiding sexual violence (Gordon and Riger, 1991; Smart, 1995; Cahill, 2001; Wendt-Höjer, 2002). In rape trials, defense lawyers often portray the complainant's behavior as irrational (e.g., ‘Why did she stay? Why did she lie down in the same bed without trousers?'). Understanding female fear can help contextualize such behaviors (e.g., staying with an acquaintance may seem safer than walking home alone at night; Wettergren et al., 2025).

Another central concept from Manne's theory can also be useful here. *Herasure* describes the phenomenon in which women's experiences and narratives, particularly those concerning sexual violence, are erased or ignored (erasing her story and experiences). This erasure can manifest through the questioning of victims' testimonies, the defamation of their character, or the simple absence of space given to their stories in legal and public discourses (Manne, 2018).

Herasure makes it more difficult for victims to seek and achieve justice, which, in turn, can deter others from reporting such crimes. In courtrooms, women who have been subjected to sexual violence are given the opportunity to recount their experiences; however, the issue of a high proportion of acquittals persists. Even under the new legislation, the proportion of convictions remains at approximately 65%, significantly lower than in most other types of criminal cases (Brå, 2023).

This can be partly understood in the light of the high evidentiary requirements, which are particularly evident in the evaluation of the complainant's testimony in these types of cases. Such requirements are tied to legal presumptions that are supported by himpathy—sympathy for men accused of rape. Ultimately, this risks leading to herasure, that is, the erasure, neglect, or diminishment of women's stories and experiences.

## Methodology

### Field observations and interviews

The methods employed in the two research projects^13^, utilizing the sociology of emotions, involved qualitative, ethnographically inspired approaches. The data was gathered through shadowing, court observations, semi-structured in-depth interviews with judges and prosecutors in both projects, and defense lawyers, and victim counsels in the project on rape. Written judgments were used in both projects. Observations, interviews, and judgments were linked to selected cases, a majority concerning rape (incl. attempted and negligent rape) but also murder, gross fraud and gross violation of women's integrity. We tracked these cases from prosecution to trial and judgment in the district court, and if appealed, in the appeals court. To identify patterns applicable across different empirical settings, cases were strategically chosen from at least three (out of six) courts of appeal and more than six district courts in various parts of the country, ensuring a balanced representation of male and female legal professionals.

Observation data and informal interviews were collected as field notes. The semi-structured indepth interviews and longer follow-up interviews were tape-recorded. Almost 30 cases yielded more than 100 interviews, including a few group interviews in the two projects. The field observations included trials and a large part of the deliberations. The analysis in this article builds mainly on the results from the project on rape cases.

Transcribed interviews and field notes have been analyzed using software for qualitative analysis. The analysis was done in two stages: open coding to organize texts into larger themes and selective coding for a focused and detailed examination of selected text segments. Codes were derived from a mix of inductive, empirically emerging concepts and deductive, theoretically informed concepts (e.g., background emotion). As empathy is the focus of this article, examples from empathic translation work performed by the legal actors in court, i.e., the prosecutor, the defense lawyer and the victim's counsel, will be at the center together with judges' empathic attunement.

## Objectivity in practice—empathic translations and dispassionate encoding

Following along the lines of foundational work by scholars such as Lynch, Latour, Scheffer, van Oorschot and Bergman Blix and Wettergren, this analysis explores embodied objectivity within courtroom settings.

Previous research on professional emotions in the courtroom has demonstrated that objectivity is not a fixed state for the judge to inhabit, but rather a dynamic and relational process continually shaped through interactions. Building on these theoretical foundations, this section explores how the concept of empathy, understood as a tool rather than merely an emotion, can facilitate perspectival translation and imagination in legal practice. By engaging with multiple, situated perspectives and exercising empathy as a means of deeper understanding, legal actors are better equipped to embody objectivity in a way that transcends traditional positivist ideals. This section will examine how such an approach, embedded in the context of himpathy and herasure, through the standpoint epistemologies suggested by Harding as part of a stronger objectivity ideal allows for a more nuanced, equitable, and context-sensitive application of the law, ultimately enhancing the impartiality and fairness of judicial decision-making.

The analysis, based on empirical material from 18 Swedish rape trials, is structured in three sections. First, the judges' perspectives on objectivity are examined, focusing on how objectivity is performed through emotional regulation and empathic engagement in the courtroom. The second section explores how entrenched gendered norms—particularly *himpathy* and *herasure*—challenge the enactment of objectivity in rape trials. It demonstrates how empathic identification with male defendants and the erasure of female complainants' experiential narratives can distort credibility assessments and reinforce structural bias. Finally, the third section turns to examples of *empathic trials*, illustrating how legal actors' use of empathic translation and standpoint epistemologies—especially through acknowledging *female fear*—can foster more equitable and context-sensitive legal reasoning.

### Objectivity and the judge

This section focuses on how objectivity is demonstrated and managed by judges. Drawing on the idea of objectivity as a collective process performed by the legal actors, as suggested by Bergman Blix and Wettergren (2018), this section offers a few examples of the professional life of the judge in rape trials, exploring how empathy may function or be used as a tool to translate everyday life into legal logics as part of the professional role in embodying objectivity.

A crucial part of collecting information during the trial in rape cases includes listening to witness statements, including the parties' stories to decide on the question of voluntariness (Swedish decisive criterion in the consent-based rape law) and intent, or negligence. Empathy may serve as a tool to tune in with others, but it has a dual function, as the judges may also use empathy to establish trust and pave way for better testimonies (Bladini and Bergman Blix, 2022).

In the following Kattis, a victim's council (from case 18) describes how judges' display objectivity and empathy. She starts in the dispassionate ideal and ends with an example of the embodiment of an empathic objective judge:

I experience many judges as uninterested. It is part of their role that they shouldn't sit and nod or agree, but rather be impartial and independent. However, one quickly notices when a judge is engaged, when they ask additional questions, when they want to clarify things, and this often stems from a type of curiosity about the clients or the case. It's like a performance—perhaps not in the Court of Appeal, but in the District Court—and when one is interested in it, it often helps the case. It also helps when the clients perceive that the judge is interested; that is the most important person, and it can mean a lot for many. Then, they also tend to share more.

They might even speak more honestly (Interview with Kattis, Victim's Council in Case 18).

The example illustrates one form of embodying objectivity through active engagement. While remaining impartial and independent, a judge can still express empathy by asking thoughtful questions and demonstrating genuine curiosity about the case. This approach reflects a balanced form of objectivity—one that does not compromise fairness but instead enhances it. By engaging empathetically, the judge encourages openness and honesty from the parties, thereby enriching the quality of the testimony and ensuring a more thorough evaluation of the facts. In this way, empathy becomes a tool that complements, rather than undermines, the judge's objective role. Furthermore, a judge builds trust and legitimacy by ensuring that laypersons feel acknowledged, heard, and treated with respect. Studies indicate that the perception of fairness during a trial is shaped more by the experience of the legal process than by the final verdict (Tyler, 2008; Leben, 2019).

However, as suggested earlier, a judge's empathic ability may also help them understand the parties' perspectives, reasoning, and rationales for action, though it is a challenging task and carries the risk that empathy could turn into sympathy. Del Mar, for example, argues that legal reasoning requires an ability to imaginatively reconstruct the situations of others, and holds that compassion can enhance this imaginative process, whereas Nussbaum's concept of the judicious spectator represents a model for how empathetic understanding can be exercised without slipping into mere sympathy. Using the analogy of reading literature or watching a play, this figure embodies the ability to place oneself in another's position while maintaining a certain detachment. When reading, one becomes deeply involved in the characters' situations, yet remains aware that it is not personal. Nussbaum, inspired by Smith, explains that this balance of empathetic involvement and critical detachment is essential for evaluating the appropriate level of emotion the participants should experience (Nussbaum, 1996). It is worth noting that Del Mar suggests using imagination to understand different perspectives, which suits well with Harding's standpoint epistemologies, arguing that several perspectives offer a more nuanced knowledge.

Below an example of when Judge Bengt from one of the rape cases discusses the troubles of evaluating the evidence in rape cases:

I've obviously thought a lot about this, and I think I can describe it as... part of it is that the stakes are unusually high. Of course, everyone who comes to court should be treated with the same respect, the same thoroughness, and so on, but in these cases, you very often have people who... have never had any previous contact with the justice system. You have a girl who has never been a crime victim before, who has never been to court, and then you have a defendant, a young man between 18 and 25, who has no prior convictions, who has never had any contact with the justice system. A medical student, or a carpenter, or a firefighter who's just starting adult life, and suddenly they are deprived of their freedom and are facing accusations which, if the prosecutor wins, as I imagine it in my head when I think about it, they will be branded for the rest of their life as a rapist. And that's a terrible burden to carry through life. You know it, ‘I'm a rapist.' And they're going to sit in prison for 2 years, or two and a half years, minus parole. On the other side, you have the victim, and I myself have two daughters in their twenties. I care just as much about them as I do about this old notion of a second violation if they don't get justice in the legal process. In this way, the human stakes are very high. Then perhaps, as a judge, it's easier to identify with these people precisely because they're first-time offenders, they could be anyone. They're people who are studying at university or could be my own children. The other day, in the evening, some old friends from the past called. Their 25-year-old son, who's studying to be a doctor, is facing... I know that these are people similar […], and that makes it feel closer. I think that this increased level of identification, as I imagine it, leads to it becoming painful... so you have the stakes for the individuals, you have the identification, but then there's also a damn difficult evidentiary situation on a more technical level. Eh... you don't have a lab report about the drugs or surveillance footage from the theft, but it's more like... yeah... like reading tea leaves.

The example from Judge Bengt's reflection illustrates the profound challenges that arise when judges must navigate the complex emotional and evidentiary landscape of rape cases. Bengt emphasizes the heightened stakes for both the accused and the victim, as well as the difficulty of remaining impartial while facing deeply human, often relatable situations. His comments align with the concept of empathetic detachment explored by Del Mar and Nussbaum. Bengt's ability to acknowledge the personal and emotional weight of such cases demonstrates the judge's struggle to maintain a balance between empathy and objectivity.

Here, empathy becomes a double-edged sword: while it aids in understanding the perspectives of individuals who have never before encountered the justice system, it also presents the risk of clouding judgment if it devolves into sympathy. This tension reflects Nussbaum's notion of the judicious spectator, who must engage empathetically with the individuals involved while preserving a critical distance. Bengt is discussing both the defendants' and the complainants' perspectives, which is in line with Hardings standpoint epistemologies. However, Bengt is highlighting the perspective of the defendant (young, not previously convicted, ruined future and the medical student (son of friends) more than the complainants (which he does by a reference to his daughters). This may be read as an expression of male fear and himpathy. By drawing on imagination and empathy, judges like Bengt can construct a more comprehensive understanding of the parties' actions and experiences, but they must also reflect upon their emotional attunement and engagement, which could compromise their objectivity when embedded in male fear and himpathy.

Furthermore, Bengt's struggle with the ‘damn difficult evidentiary situation' highlights another critical point: empathy alone cannot resolve the technical challenges of legal reasoning. As Harding's standpoint epistemologies suggest, multiple perspectives provide a more nuanced understanding of truth, but this must be carefully balanced with rigorous, evidence-based analysis. Judges must therefore navigate a complex terrain, where both empathy and detachment are necessary tools for achieving justice. In rape cases, particularly, this balance is key to ensuring that all voices are heard and respected, while still adhering to the principles of fairness and impartiality. The difficult evidentiary situation, combined with the burden of proof, high evidentiary threshold (beyond reasonable doubt) and the presumption of innocence is also strengthening the tendency to tune in with him (himpathy).

In conclusion, Judge Bengt's reflections underscore the importance of integrating empathetic understanding into judicial reasoning but also shows the need for detachment through critical reflection. The ability to imaginatively engage with the parties involved, as advocated by Del Mar, can humanize the legal process, but it must be tempered by the judicious spectator's cautious, critical stance to ensure that justice is served both compassionately and fairly.

### Trials troubling objectivity

Following the arguments of Lynch, the *embodiment* of objectivity occurs through routinized actions—like the handling of DNA evidence or the structured questioning of witnesses—which bring about what we consider ‘truths' within the context of legal reasoning. He argues that the production of objectivity is thus tied to procedures, routines, and performances that lend credibility to certain knowledge claims. Lynch et al. (1995) draws on the idea of *embodiment* to suggest that scientific truths do not just exist as abstract entities but are enacted by human bodies engaged in material practices. In a legal setting, for instance, forensic scientists, lawyers, and even courtroom participants (judges, lay judges) all play roles in giving life to facts through their embodied actions. Rape cases are often characterized as ‘word-against-word' situations, seemingly lacking hard (technical or forensic) evidence. However, this is not entirely accurate, as such cases frequently include various forms of technical evidence, such as DNA, text messages, and other digital traces (Wettergren et al., 2025; Smith, 2018). The challenge, however, lies in the fact that the key evidence regarding consent and intent typically remains oral testimony.

These situations, as previously discussed, highlight the importance of empathetic translation. However, they are also embedded in norms and assumptions about male and female sexuality, the “real” rape, and the ideal victim, all of which pose a particular risk for himpathy, i.e., when empathy turning into sympathy, particularly for the defendant. As shown by Uhnoo et al. (2024b), this may partly be explained by a worry to be too engaged in the complainant's perspective. This can occur when judges or other legal actors overly identify with the defendant's background or circumstances, like in the quote from Judge Bengt above.

#### Empathy shifting into himpathy

In the courtroom, judges and other legal actors are expected to maintain objectivity, navigating the emotional dynamics of a trial without allowing personal biases to interfere with the legal process. However, as Kate Manne argues in her work on gendered dynamics of sympathy, there is a notable tendency for empathy to shift into himpathy—the term Manne coined to describe the societal inclination to sympathize with men who are accused of sexual misconduct or assault. Himpathy refers to the disproportionate concern and empathy shown toward men, especially when they are perceived as being at risk of losing their reputation, career, or freedom due to allegations of sexual misconduct (Manne, 2018).

This phenomenon is particularly evident in cases involving young, previously un-convicted men, where the courtroom actors, including judges, may be drawn into a sympathetic understanding of the defendant's plight. This often manifests in statements such as “it could have been my son,” where legal actors express concern for the young defendant's future, thus centering the trial on the impact the accusation may have on his life, rather than on the victim's experience (cf. Uhnoo et al., 2024b).

For example, in one of the case interviews, *Judge Bengt* reflects on the challenges of dealing with young defendants who face life-altering accusations: “*You have a young man, unconvicted, on the brink of adulthood, suddenly facing accusations that could brand him a rapist for life. It's a terrible burden to bear, and it's not surprising that this identification leads to sympathy.”* Such expressions show how empathy for the defendant's potential loss of freedom and future prospects can overshadow the gravity of the crime itself and the harm caused to the victim.

There are also many examples of how the defense lawyer successfully describes the defendants' behavior as rational and logic, or if irrational, explained by a male fear defense. For example, when defendants denies everything during the first hearing, at an early stage of the investigation. He may deny that he knows the complainant at all, or denying that they have met, or at least denying that they have had intercourse. Then, when DNA evidence shows that they (the complainant and the defendant) did have intercourse, the defendant suddenly changes his story into a consent-defense. This change of statement is usually explained by the defense lawyer as a natural reaction due to male fear. The shared male fear of being innocently accused for rape or other sexual violence. The immediate reaction to deny is then logic and rational (cf. Uhnoo et al., 2024b).

#### Herasure: erasing the victim

In contrast, the victim's memories and experiences are often diminished or erased—a phenomenon Manne refers to as herasure. This describes the way in which women, especially victims of sexual violence, are rendered invisible in the narratives that arise during trials, as the focus shifts toward preserving the reputation and future of the accused (Manne, 2018). Herasure can be seen in courtrooms when victims' stories are dismissed as unreliable or when their trauma is downplayed in favor of a more sympathetic narrative toward the defendant. This erasure can further marginalize the victim, who may already struggle to articulate her experience within a legal system that demands objectivity but often fails to recognize the emotional and social contexts of sexual violence.

This dynamic has been explored through the lens of courtroom discourse, where the victim's testimony may be seen as less credible or is sidelined in favor of more “rational” or “logical” accounts that privilege the defendant's narrative (cf. Uhnoo et al., 2024b). This aligns with the idea that in many cases, the victim's emotional response is seen as less legitimate or too subjective, reinforcing a legal culture where women's experiences of trauma are systematically diminished.

For instance, in one case, a defense lawyer argued that the victim's inability to recall certain details was a sign of unreliability, whereas the defendant's shifting storylines were excused as the result of shame or confusion.

She's not able to recall the precise events, which affects the reliability of her statement. Meanwhile, my client is young and deeply embarrassed about the situation, which is why he initially withheld details.

This highlights the tendency to cast the victim's uncertainty in a negative light, while offering more leniency toward the defendant's inconsistencies, further contributing to the erasure of the victim's perspective in the pursuit of a sympathetic narrative for the accused.

In light of these dynamics, trials involving accusations of sexual assault can reveal the tension between maintaining objectivity and the subtle ways in which empathy can shift into *himpathy* or lead to *herasure*. Legal actors must navigate this delicate balance, as empathy for the defendant's potential hardships should not result in a diminished focus on the victim's experience or the seriousness of the crime. Yet, as evidenced by both Manne's theoretical framework and the findings in Uhnoo et al. (2024b), this balance is often difficult to maintain, especially when societal norms continue to favor protecting the interests of men accused of sexual violence.

### Empathic trials

In one case (case 11), the female victim allowed her male friend and his friend to stay overnight as they were on a long journey and lived far away. Although she expected two guests, six people, including four unknown women, arrived in the middle of the night, drunk and wanting to continue partying. Upset, she asked them to leave, but one man (her friend's friend) requested to use the bathroom, and she let him in while the others left. Alone with this drunk stranger, she let him stay, lent him jogging bottoms, and offered him a bed. A crucial legal question was whether this situation could be interpreted as an invitation leading to voluntary sex.

During the hearing, legal professionals created empathy to help judges understand the parties. The defense lawyer questioned the victim: “So he took off his clothes and lay down on your bed? And you didn't ask him to leave?” The victim replied that she texted her friend for help but didn't tell the defendant, instead asking him to move to the spare bed. The defense lawyer framed her behavior as irrational: “This sounds a bit strange to me. You are telling us that you slept, woke up, got a kiss from [the defendant] that you didn't ask for, but you didn't ask him to leave?” The prosecutor countered, arguing her reaction was reasonable given her emotional involvement with the defendant's friend, suggesting she tried to protect that relationship by seeking help discreetly.

The victim's counsel frames her actions as “correct (normal, natural, reasonable and rational)” by referring to common responses among women fearing sexual assault. She asserts:

”If a male stranger got into my home, I would have acted with caution. I'm not so sure a woman would dare to resist, if she was unsure if she would be able to overpower the other person. As women, we always fear sexual assault. Wherever we are and wherever we go. Her way of dealing with the situation was completely correct.”

This plea illustrates the struggle between legal actors over defining rational and logical behavior in specific situations. The defense lawyer seeks to depict the complainant's actions as irrational or inconsistent with the behavior of someone not interested in sex, thereby undermining her credibility. In contrast, both the prosecutor and the victim's counsel work to render her actions comprehensible and situationally rational, aiming to establish that her participation was not voluntary.

The District Court hearing exemplifies the interpretive struggle over voluntariness and credibility, where legal actors engage in empathic translation, mobilizing different frameworks to either support or discredit the complainant's narrative. The defense lawyer relies on normative assumptions and portrays her actions as illogical, thereby casting doubts on the veracity of her narrative. Meanwhile, the prosecutor and the victim's counsel strive to translate her actions and behavior to be intelligible and coherent, framing it in a way that affirms her credibility. The victim's counsel, in particular, explicitly draws upon a logic of female fear to explain the rationality behind the complainant's actions.

In this context, empathy becomes a crucial interpretive tool, enabling legal actors to navigate shared experiences and memories that differ by gender. The dynamic between the female victim's counsel and the male prosecutor and defense lawyer underscores how gender mediates the interpretation of actions and intentions. Empathic understanding, grounded in standpoint epistemologies, plays a key role in evaluating testimony and determining voluntariness—particularly when conventional legal reasoning may fail to account for gendered logics.

In many trials, defense lawyers attempt to depict the complainant's behavior as irrational, abnormal and thus untrustworthy, just like in the example above. Common strategies include posing questions such as: *Why did you stay at an older acquaintance's home in the middle of the night? Why did you not call a taxi that late evening? Why did you not take a tram home at 4 a.m.?* All of these questions can be answered through the lens of female fear logics: it may appear safer to remain in a private space with someone vaguely familiar than to risk exposure to potential threats in public spaces (such as a deserted tram stop) or with unfamiliar men (such as a taxi driver). However, unless this logic is made explicit, the complainant's actions may be perceived as irrational.

Additional questions posed by defense lawyers often follow a similar pattern: *Why did you sleep in the same bed if you did not intend to have sex? Why did you remove your trousers before going to bed next to someone you were not sexually interested in?* These questions rely on normative assumptions rooted in a male-coded logic of sexuality and consent, where physical proximity or partial undressing is interpreted as evidence of sexual interest. Unless this logic is challenged and placed in the context of female fear—which may inform seemingly contradictory behavior—such questions risk undermining the complainant's credibility by rendering her actions irrational. One strategy observed among young women to navigate or resist unwanted sexual expectations is the performance of everyday normalcy, such as watching a film together even in the same bed under a shared blanket. This can be understood as an attempt to maintain social coherence while avoiding escalation into a sexualized situation (Wettergren et al., 2025 in print).

Moreover, such lines of questioning may contribute to forms of testimonial injustice, whereby the complainant's account is dismissed or devalued, especially when the adjudicating perspective lacks access to, or fails to acknowledge, the gendered standpoint from which the complainant speaks.

Such moments of empathic translation are central to what may be described as *empathic trials*—legal proceedings in which all actors engage in efforts to understand, translate, and legitimize the experiential logic of the complainant. In cases involving sexual violence, this often requires the prosecutor and the victim's counsel to actively foreground a standpoint shaped by *female fear*, making visible the embodied risk assessments and protective strategies that underpin seemingly contradictory behavior.

Through such translation work, they counteract the effects of *himpathy*—the tendency to empathize with male defendants and dismiss the credibility of female complainants—and instead contribute to a more balanced and context-sensitive assessment of guilt. In this way, empathic trials not only facilitate procedural fairness, but also create space for epistemic justice within the courtroom.

## Conclusion

This article set out to explore how empathy functions within legal proceedings, particularly in rape trials, and how it interacts with judicial ideals of objectivity. While recent legal reforms in Sweden aim to strengthen women's bodily and sexual integrity—most notably through the introduction of consent-based legislation—these efforts unfold within a legal and societal context still deeply embedded in structures of *himpathy* and *herasure*. These embedded norms risk undermining the practical impact of reforms by framing women's actions as irrational or unreliable and by disproportionately empathizing with male defendants. Against the backdrop, the article has demonstrated that objectivity is not undermined by empathy; rather, it is co-produced through emotional management and empathic translative and interpretive labor among legal actors (c.f. Bergman Blix and Minissale, 2022; Bladini and Bergman Blix, 2022).

Empathy emerges as a critical epistemic tool that enables judges and other legal professionals to grasp the situated rationalities of those appearing before them. The study shows that legal actors engage in *empathic translation* to render the complainant's actions intelligible within a legal framework—particularly as many of those actions are shaped by gendered logics. Such translation work must therefore be grounded in gendered standpoint epistemologies, recognizing that actions shaped by fear, risk awareness, and social vulnerability are intelligible only when viewed from within the situated knowledge frameworks of those who live them. This translation work is vital for countering forms of *testimonial* and *hermeneutical injustice*, ensuring that credibility assessments are not distorted by normative assumptions about rationality and behavior.

The courtroom is revealed as a site of emotional labor and epistemic negotiation, where objectivity is enacted collectively—through narrative framing, embodied responsiveness, and affective attunement. Rather than compromising neutrality, empathy strengthens the foundations of a more *reflexive* and *inclusive* legal process. Legal actors, including prosecutors and victim's counsels, play a particularly important role in making visible the complainant's standpoint, especially when her actions reflect the logic of *female fear*—a perspective that might otherwise be dismissed as irrational or inconsistent with voluntariness.

Building on Sandra Harding's concept of *strong objectivity*, this article demonstrates how standpoint epistemologies can be operationalised in legal settings (Harding, 1992; Bladini, 2013). The gendered logics of *female fear* and *male fear* function here as situated epistemic standpoints that shape how actions and intentions are interpreted in rape trials (Wettergren et al., 2025). Rather than viewing objectivity as detachment, Harding's framework—as applied in this study—emphasizes reflexivity, positionality, and the inclusion of marginalized experiential knowledge as necessary conditions for more just and reliable legal reasoning.

This argument is further supported by Bergman Blix and Wettergren's (2018) conceptualization of the *doing of objectivity* as an emotionally regulated practice, and by Del Mar's (2017) notion of *imaginative legal reasoning*, which recognizes the central role of empathy and perspective-taking in judicial work. Together, these perspectives challenge traditional positivist ideals of legal reasoning and offer an alternative model grounded in emotional engagement, contextual sensitivity, and epistemic plurality.

While the shift to consent-based legislation in Sweden aims to address the structural roots of sexual violence and to safeguard the sexual autonomy of women, this study suggests that deeply embedded legal assumptions continue to pose challenges. Normative logics aligned with *himpathy* and *male fear* may be subtly reinforced through principles such as the presumption of innocence and the high burden of proof. Although these principles are foundational to criminal justice, they may also obscure the lived realities of complainants unless counterbalanced by empathic, gender-aware interpretation (Wettergren et al., 2025).

Ultimately, this article argues for a more embodied, relational, and reflexive model of objectivity in legal practice—one that recognizes empathy not as a threat, but as a condition for fairness. In cases of sexual violence, where fear, power, and asymmetries of experience shape every aspect of testimony and interpretation, such an approach enables a more human-centered and epistemically just legal process.

## Data Availability

The data analyzed in this study is subject to the following licenses/restrictions: the article consist of three datasets, the first public material from legal doctrine, legal documents and judgments. The second and third from two research projects, one finished and one not yet finished. The data collection, storage and analysis is described in the article. Requests to access these datasets should be directed to moa.bladini@law.gu.se.

## Footnotes

^1^Weber interpreted the development of modern law as intertwined with the processes of rationalization and bureaucratization. He distinguished between substantive law, which is influenced by ethos and emotion, and formal law, which operates on objective instrumental reason and administers justice impartially, treating all individuals equally and ‘without regard to persons' (Weber, 1998: p. 214).

^2^ https://eige.europa.eu/publications-resources/publications/gender-equality-index-2024-sustaining-momentumfragile-path

^3^Regeringsformen [The Instrument of Government] (RF) Chapter 1 Section 9; Chapter 2 Section 11.

^4^Article 6 in ECHR and article 47 in the Treaty of Lisbon.

^5^Rättegångsbalken [Swedish Code of Judicial Procedure] (RB) Chapter 4, Section 11.

^6^Rättegångsbalk [Swedish Code of Judicial Procedure] (RB) Chapter 30, Section 3.

^7^These principles are not unlimited; the question of admissibility is constrained by considerations such as costs and efficiency, while the evaluation of evidence is guided by both legal doctrine and jurisprudence from the Swedish Supreme Court. While the Supreme Court in Sweden traditionally engaged in legal questions, but not evidential issues, this has changed since the 1980's.

^8^Further reading on the Swedish system with lay judges, see e.g. Diesen (1996); Roos (2022).

^9^Rättegångsbalk [Swedish Code of Judicial Procedure] (RB) 23:4 and 45:3 a.

^10^The role of the victim's counsel is somewhat ambiguous in relation to the prosecutor, see Wettergren et al., 2025.

^11^The turn came with the research of the neuroscientist (Damasio, 1994). His studies on people who lost contact with their emotional parts of the brain, but had their cognitive capacity intact, after suffering from brain damage shows that these people had problems with decision making. Either they made risky decision or they got stuck in the process of reasoning: ‘on the one had… but on the other hand…' the result of Damasio's analysis was that emotions are necessary for the ability to make decisions in the sense that one needs to feel the consequences of one's actions.

^12^Del Mar's concept of *perspectival imagination* aligns with Harding's standpoint epistemology by offering a practical means for judges to navigate multiple perspectives without compromising impartiality. Harding's idea that knowledge is socially situated underscores the need to recognize marginalized viewpoints, while Del Mar emphasises the importance of imagining diverse perspectives, including hypothetical ones, to avoid privileging any single narrative. This *imagining by feeling*, as Del Mar calls it, not only supports impartiality but is essential to it, presenting an embodied approach to objectivity where empathy serves as a tool for understanding, rather than a source of bias. By synthesizing these ideas, I analyse how legal actors can move toward a more embodied objectivity in practice.

^13^*Rape or consent - Effects of the new rape laws on legal reasoning and practice* (RJ P19-0515:1) and *Construction of Objectivity* (VR 2016- 01218).
